# modelRxiv: A Platform for the Dissemination and Interactive Display of Models

**DOI:** 10.1111/ele.70042

**Published:** 2024-12-31

**Authors:** Keith D. Harris, Guy Hadari, Gili Greenbaum

**Affiliations:** ^1^ Department of Ecology, Evolution and Behavior The Hebrew University of Jerusalem Jerusalem Israel

**Keywords:** computational modelling, eco‐evolutionary dynamics, modelling, modelling frameworks

## Abstract

Modelling the dynamics of biological processes is ubiquitous across the ecological and evolutionary disciplines. However, the increasing complexity of these models poses a challenge to the dissemination of model‐derived results. Often only a small subset of model results are made available to the scientific community, with further exploration of the parameter space relying on local deployment of code supplied by the authors. This can be technically challenging, owing to the diversity of frameworks and environments in which models are developed. To address this issue, we developed a platform that serves as an interactive repository of biological models, called modelRxiv. To facilitate adding models to modelRxiv, we utilise large‐language models (LLMs) to make the platform language‐agnostic. The platform provides a unified interface for the analysis of models that do not require any technical understanding of the model implementation, thus improving the accessibility, reproducibility and validation of ecological and evolutionary models.

## Graphical abstract

modelRxiv is an interactive repository of eco‐evolutionary models that enables readers to access published and unpublished models without the technical hurdles of understanding model code. The platform integrates LLMs to streamline the addition of new models, making it programming language‐agnostic.

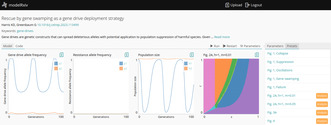

## 1 Introduction

Modelling the dynamics of ecological and evolutionary processes has been key to the development of ecology and evolution for more than a century (Otto and Day 2007). Over the past few decades, the availability of computational power has paved the way for models of increasing complexity, involving numeric, stochastic and individual‐based modelling features, and exploration of multiple variables spanning over large parameter spaces (Grimm and Berger 2016). Detailed and high‐resolution investigation of models can offer important insights on general biological phenomena, as well as for specific systems. However, traditional scientific publishing standards—static papers depicting several model results representing select parameter values—are limited in demonstrating the full scope of model results and their implications. This affects both the dissemination of potential insights from a model and the ability to assess the robustness of model results during the scientific peer‐review process.

Currently, models in ecology and evolution, and across the biological disciplines, are implemented in a number of different programming languages because there is no standard framework for publishing model implementations. Consequently, deploying models locally by a reviewer or a reader usually requires prior knowledge of the specific coding language or framework in which the model code was written, the installation of dependencies (e.g., code packages), and learning how to operate the model code and visualise results. This technical hurdle can make published models essentially inaccessible, where only the authors of the model are able to reproduce its results and alter the model.

Various platforms have been developed to address some of these difficulties. These approaches can be categorised into two broad classes: (i) a ‘model‐centric’ approach, where the interface is focused on model analysis and visualisation, and users are not required to view, edit or understand the underlying model code, and (ii) a ‘code‐centric’ approach, where users manipulate model code in order to analyse the model. Model‐centric platforms usually provide a single programming language for modelling (e.g., *NetLogo* (Tisue and Wilensky 2004), *Mathematica* (Wolfram 1991), *MATLAB* (MATLAB 2021), *shiny* (Chang et al. 2024)), while code‐centric platforms accommodate the deployment of multiple programming languages in a single interface (e.g., *Jupyter* (Kluyver et al. 2016), *CodeOcean* (Staubitz et al. 2016)). In both classes, models are accessed through a unified interface, reducing the investment needed by reviewers or readers to interact with the model. However, most published models are implemented in different programming languages, and translating model code so that it is compatible with a model‐centric platform can be impractical. On the other hand, code‐centric solutions that support multiple programming languages are focused on model implementation and lack features that can make model analysis straightforward and accessible.

To bridge the gap between these two approaches, we developed an interactive repository of biological models called modelRxiv (https://modelrxiv.org). Our web‐based platform allows users to visualise the results of many different types of published and unpublished models from the same interface without having to download or install framework‐specific software. modelRxiv is designed with a simple user interface that does not require a technical understanding of programming or modelling to operate. To streamline the implementation of this protocol for existing models, and the translation of these models to browser‐compatible programming languages, we provide an AI‐assisted process of adapting the model code to the modelRxiv platform. We envision this platform as a tool for model exploration by reviewers and readers, and as a repository that can make published and unpublished models easily accessible to the scientific community.

## 2 Platform

**FIGURE 1:**
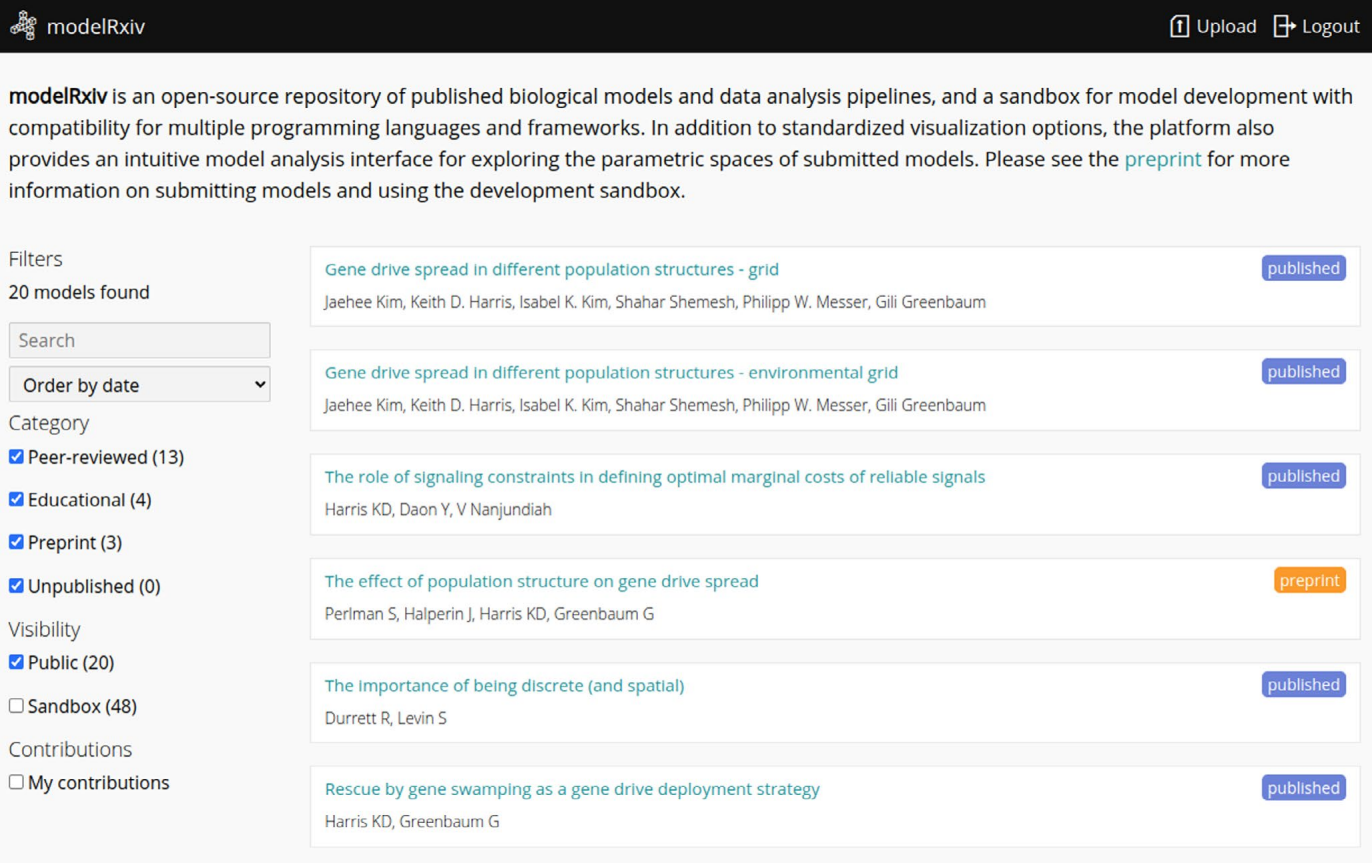
The main page of modelRxiv, which lists available models on modelRxiv belonging to different categories. On this page users can search and sort models, and select a model to view its model analysis page. When authenticated, the index will list models that are publicly available, as well as private models (‘Sandbox’ models) uploaded by the user.

**FIGURE 2:**
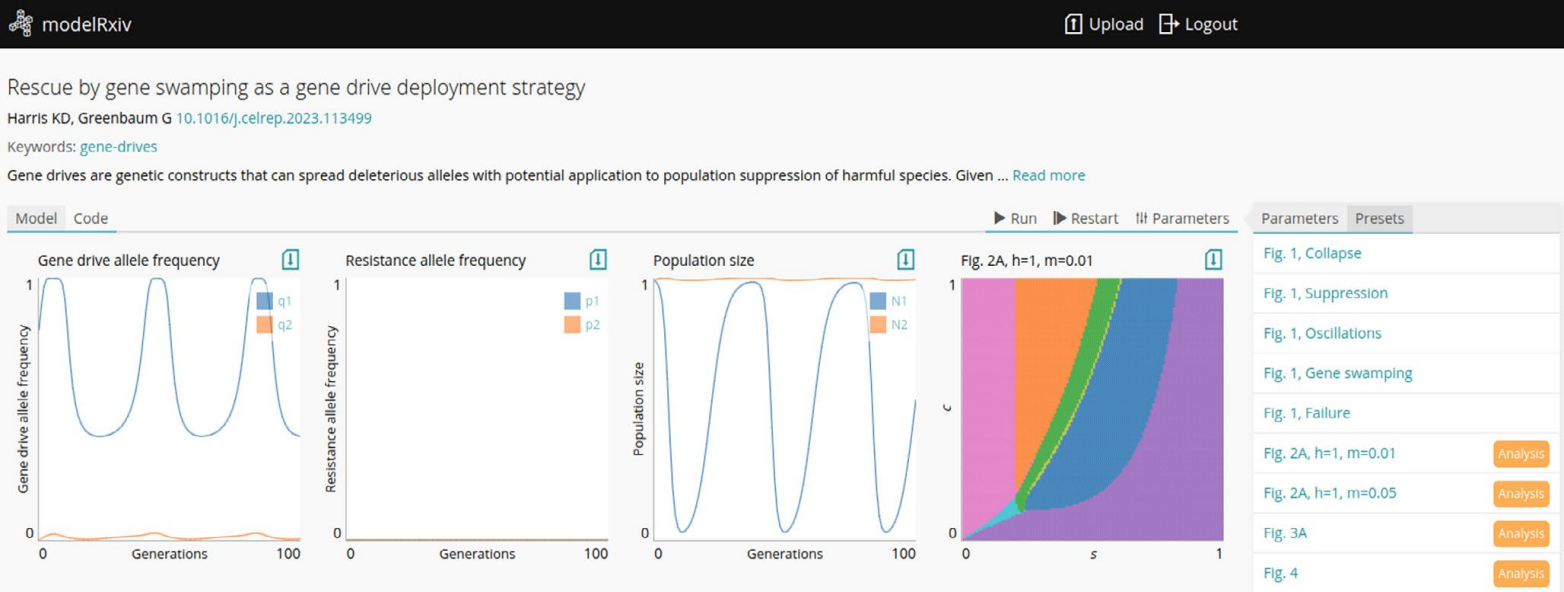
Dynamics of gene drive model generated on‐the‐fly (Harris and Greenbaum 2023). At the top of the page, metadata relating to the model is shown, including author information, the title and description of the model and a link to the manuscript if available. Underneath are the dynamics that have been generated on‐the‐fly: in this case, the plots include the genetic and demographic dynamics of the gene drive model (the first three plots). The fourth plot is an ‘analysis’ that has been run by pressing one of the presets in the ‘Presets’ menu to the right of these panels. This menu allows users to reproduce specific dynamics of the model, for example, replicating the figures in the manuscript, or running various analyses (indicated with the orange tag ‘Analysis’).

**FIGURE 3:**
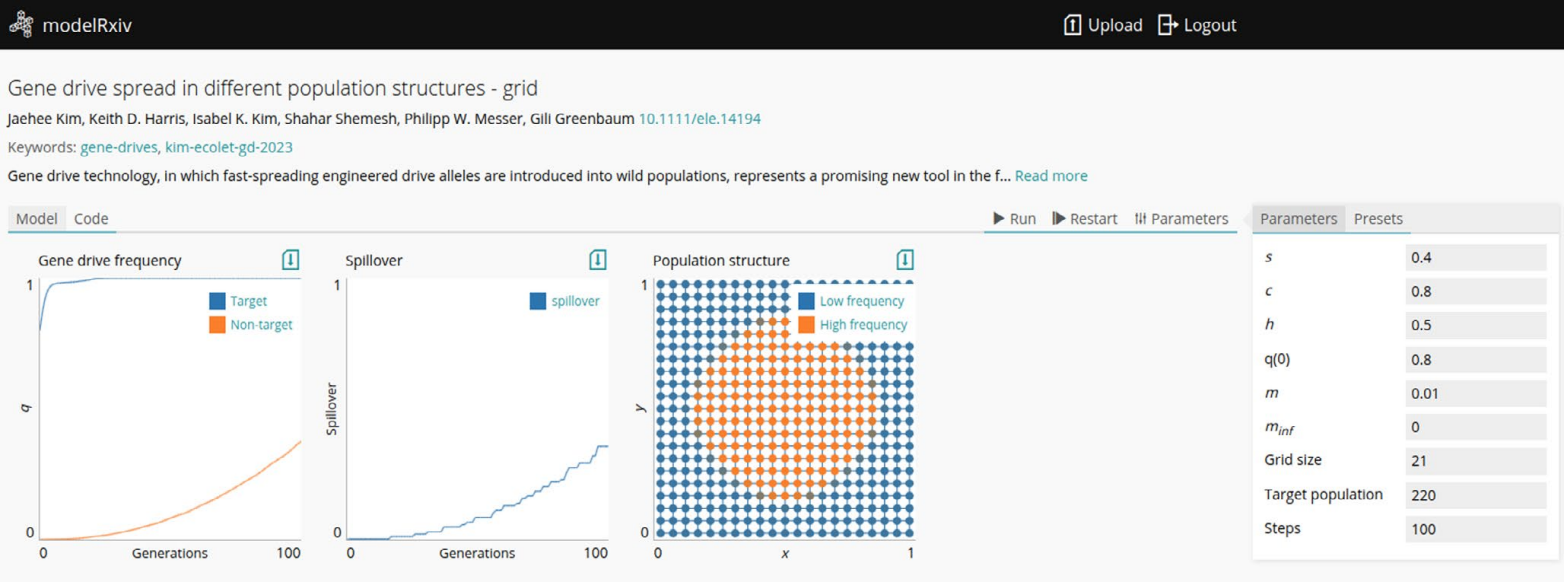
Spatial dynamics of a gene drive model that incorporates population structure and dispersal (Kim et al. 2023). The spread of the gene drive through populations is visualised as nodes with the colour reflecting the frequency of the gene drive in each population (right‐most plot on the left panel). Users can track the overall spread as the number of populations above a certain threshold (the ‘Spillover’ panel) or observe the spatial dynamics (the ‘Population structure’ panel). These standardised displays can be used for many different types of models. Users can manipulate the parameters listed on the right and rerun the model dynamics to observe the effect of different parameter configurations on the result.

**FIGURE 4:**
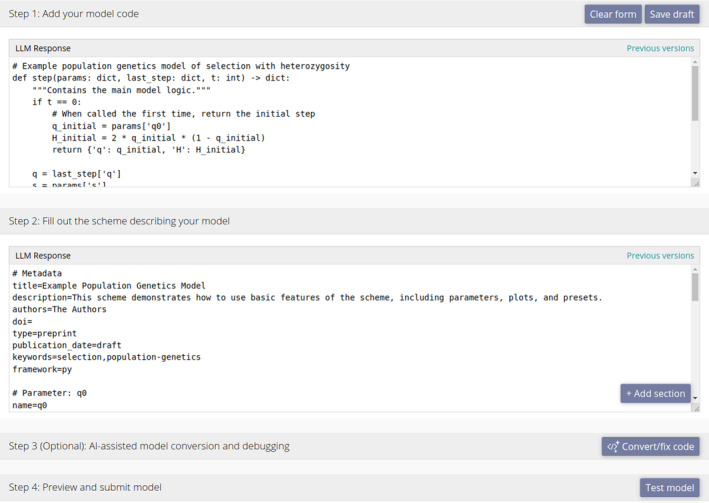
Upload interface showing an existing model code and scheme. The form is divided into four steps: (i) providing the model code, (ii) creating a scheme describing the model, (iii) an optional AI‐assisted conversion step and (iv) testing the model is compatible. In this example, the model code and scheme were completed correctly by an LLM. The response of the LLM replaces the current code and scheme; previous versions of the scheme or code can be loaded by clicking on the version in the ‘Previous versions’ menu on the right.

modelRxiv is browser‐based, with model computation and visualisation occurring in the browser. The modelRxiv user interface has three main screens: (i) an index of models that can be filtered and sorted according to model metadata such as title, authors, model categories and keywords (Figure 1); (ii) model analysis pages, which include model visualisation, regeneration of predefined model dynamics and parameter manipulation features (Figures 2 and 3) and (iii) a model upload page for uploading and updating models (Figure 4). Models can be either ‘public’, viewable by everyone, or in ‘sandbox mode’ where only the owner can see and interact with the model. Public models can be viewed and analysed without logging in or creating a user, but registration is necessary to upload models and analyse models in sandbox mode (registration is free and anonymous). In this section, we describe the main features of modelRxiv that can be accessed through the model analysis page or the model upload page. For a discussion of the current limitations of the platform, and an indication of the types of models that can currently be uploaded, see the ‘Limitations’ section.

In order to explain the features of the platform, we refer to published models (Kim et al. 2023; Harris and Greenbaum 2023) that have been uploaded to modelRxiv that relate to gene drives (a genetic element that violates Mendelian inheritance) (Bier 2022). This field is an interesting use case for modelRxiv, as having a repository of published gene drive models in a single platform could be instrumental in developing direct interactions between modellers and regulators (Long et al. 2020; Combs et al. 2023; Frieß et al. 2023).

### 2.1 Model Analysis Page

The model analysis page provides users with basic model metadata, such as publication information, as well as interactive figures that are generated on‐the‐fly by the user (Figure 2). Users can interactively run model dynamics that will be visualised according to the type of output generated by the model (e.g., line plots for continuous variables, two‐dimensional grid for visualising spatial dynamics). They can also generate additional, static figures if such analyses were defined by the model uploader (see the ‘Uploading models’ section below).

Model exploration can be conducted either by regenerating results of parameter sets specified by the model authors, or by specifying custom parameters. Models uploaded to modelRxiv can have multiple parameter ‘presets’, meaning parameters that relate to a certain scenario of interest in the model (Figure 2). These can be linked to a figure in a published manuscript, allowing users to replicate published figures, and continue exploring the model by examining the effect of changing model parameters while starting off the exploration from the published parameters. Presets make model results more accessible to users, by guiding them to specific parameter sets that generate dynamics of interest. The presets menu is opened by default on the right side of the page if presets are provided by the authors. Clicking on preset labels will run model dynamics for the specified preset. Users can also stop or restart the dynamics using the control buttons at the top right corner of the model analysis page.

The presets menu can also include figures that do not involve an iterative process suitable for plotting in the model dynamics. These presets are called ‘analyses’, and can involve more intensive computation because their visualisation is static. In the gene drive model presented above, several analyses have been defined as seen in the presets menu (Figure 2). Clicking on these analysis‐type presets will run the predefined analysis and generate a single figure output. In the example in Figure 2, a fourth plot has been generated, showing types of outcomes of the gene drive dynamics for a subset of the parameter space of possible model parameters.

Model dynamics can be run by clicking the ‘Run’ button, and can be stopped by clicking ‘Pause’. Clicking ‘Restart’ will end the current model dynamics and restart with the currently selected parameters (Figure 2). If users wish to generate additional visualisations of the dynamics, or aggregate outputs, results generated by the model analysis page can be exported as CSV files by clicking the ‘Download’ icon at the top right corner of the plot.

In addition to running parameter presets, users can manipulate model parameters and rerun the model, testing the effect of different parameter values on the results (Figure 3). Clicking the ‘Parameters’ tab on the right side of the model analysis page will open a list of parameters that are available and can be modified; after changing parameter values, users can rerun the model dynamics by clicking ‘Run’ or ‘Restart’ (Figure 3). Analyses will also be run with the updated parameters. For instance, in the previous example (Figure 2), the user can change the parameters of the model and regenerate the analyses to test the effect of different parameters on the figures.

### 2.2 Uploading Models

This section describes the process of uploading models to modelRxiv. For a comprehensive guide for uploading models, including examples and screenshots for each visualisation type, see the guide for uploading models on the ‘Help’ page of the modelRxiv website (see https://modelrxiv.org/help.html or Data S1). To allow flexibility in the integration of models with modelRxiv, we developed a language‐agnostic protocol for model inputs and outputs. This protocol requires that the code implements a step function in order to integrate with modelRxiv, and a text file describing the model and its parameters (the model ‘scheme’). In addition to visualising model dynamics, the model can also implement ‘analyses’, which are functions that produce a figure that can be presented to the user.

One of the features of modelRxiv is that model computation takes place in the browser environment. This means that model code needs to be implemented in either JavaScript or Python, which are compatible with this environment. Recognising that this requirement might introduce hurdles for some users, and to make the platform language‐agnostic, we developed an AI‐assisted conversion pipeline to facilitate the conversion of models to the modelRxiv protocol, and to Python if not already implemented in JavaScript or Python. This pipeline uses a large‐language model (LLM) provided with comprehensive instructions and examples of compatible models (for details, see the ‘AI‐assisted conversion pipeline‘ section of the Methods).

While we aimed to accommodate many different types of models in the design of the protocol and the conversion pipeline, the platform is currently less suited for models that require long‐running analyses or include extensive original code libraries. See the ‘Limitations’ section for a discussion of the current limitations of the platform.

To streamline the process of uploading models to modelRxiv, we developed an upload interface that allows users to upload their code and test whether it is compatible with modelRxiv, and whether the graphical outputs are correct. The upload form is accessible to authenticated users by clicking the ‘Upload’ button at the top right corner of the screen. The process is separated into four steps (shown in Figure 4): (i) providing the model code, either in a format that fits the modelRxiv protocol, or to be converted using the AI‐assisted conversion pipeline; (ii) a scheme describing the model metadata, parameters, plots and presets, which can also be generated using the conversion pipeline; (iii) an optional step of using the AI‐assisted conversion pipeline (this box also shows textual responses from the LLM, and allows users to manually add comments to be sent to the LLM along with their code) and (iv) an interface for testing the model, which attempts to generate the model dynamics and reports on errors in the code or scheme. After completing these steps, the user can save the model as a ‘sandbox’ model using the ‘Save model’ button.

The AI‐assisted conversion pipeline utilises OpenAI large‐language models LLMs (OpenAI 2023, 2024). This feature is made feasible by the simple design of our model protocol, as well as the syntax of the model scheme, which is designed to balance readability by users and LLMs and be easily processed by modelRxiv. To utilise these features, users can provide code that is not yet compatible with modelRxiv into the code box (‘Step 1’ in Figure 4). It is preferable to add information to the model scheme prior to conversion, such as the description of the model, and details of parameters the purpose of which cannot be understood from the code alone, as this will improve the accuracy of the conversion process. To convert the model, users click the ‘Convert/fix code’ button (Figure 4). This sends the model code and scheme to an LLM along with instructions on making them compatible with modelRxiv, and examples of different models and plots (for details, see the ‘AI‐assisted conversion pipeline‘ section of the Methods). The result can then be tested from the upload page using the ‘Test model’ button. If the test fails, users can send the code along with the error to the AI assistant; this will generate a new version of the code or scheme addressing the error. In addition to technical validation of compatibility, model dynamics should be manually validated against the original outputs to assure that the model has been imported correctly. If the dynamics do not appear correctly, users can add comments for the LLM inside the panel of ‘Step 3’, which will open when the conversion pipeline is run.

After successfully testing the model, users can save the model by clicking the ‘Save model’ button. Models are visible and accessible only to the account owner and appear as ‘Sandbox’ models. This gives users the ability to ensure that the model operates correctly before submitting it to the public modelRxiv repository. Uploaded models can be edited using the ‘Edit’ button on the model page. Model code and parameters are versioned so that previous versions can be browsed and restored if necessary. To submit the model to the public repository, users click the ‘Publish’ button on the model analysis page. This will transfer the model to a moderator who will review the model manually to ensure it is valid, and subsequently the model will be publicly available on modelRxiv.

## 3 Discussion

Here we presented and described the features of the modelRxiv platform, an interactive repository of models. The purpose of the platform is to facilitate reviewers and readers in evaluating and investigating models, to increase the accessibility of published and unpublished models in ecology and evolution, and to serve as an educational tool. To allow a common interface for multiple types of models, we developed a language‐agnostic protocol for adding models to modelRxiv, and integrated LLMs into the process of adding models to the repository. Using instructions designed to increase the successful conversion of models to the modelRxiv protocol, these LLMs are able to adapt models written in various programming languages (Table 2). This interface includes validation of model code for compatibility errors. These features remove many of the technical hurdles that can be encountered in adapting code to specific frameworks of model‐centric platforms.

In our development of the model uploading pipeline, we found current LLMs capable of adding a variety of programming languages to modelRxiv without introducing errors in the code. In a rudimentary analysis, we show that LLMs designed for reasoning were able to convert multiple programming languages to Python compatible with modelRxiv in a single attempt, with high success rates (Table 2). The successful integration of LLMs with modelRxiv relies on a number of features of our platform: (i) the model protocol is straightforward and identical for all model types; (ii) the model scheme syntax is robust to typographical errors and can be read and generated by LLMs; (iii) using Python as a default language makes model conversion simpler since LLMs contain extensive Python‐related information in their training data and (iv) models can be validated in terms of their compatibility with modelRxiv owing to the use of unified outputs. LLMs are particularly suited to cases where the output pattern has well‐defined guidelines that can be enhanced by providing previous examples. Thus, modelRxiv is a perfect use case for LLM‐assisted coding. In addition, our platform will be able to use the growing repository of models in modelRxiv to fine‐tune the AI‐assisted conversion of models. The field of LLMs is rapidly developing, and modelRxiv can directly benefit from this, by adopting newly released LLMs that provide improved conversion performance.

With the development of new platforms such as modelRxiv, it is important to evaluate and compare the main features to existing platforms to better understand their potential contribution. There are a number of established comparable platforms that provide an interface for manipulating model parameters and visualising dynamics or results, such as *Mathematica*, *MATLAB*, *Jupyter* and *NetLogo*. When designing modelRxiv, we considered the features already offered by these platforms, summarised in Table 1.

**TABLE 1:** Features of modelRxiv compared to other platforms that are used for modelling.

Platform	Approach	Functionality	Computation in browser?	Language‐agnostic?
modelRxiv	Model‐centric	Repository/Analysis	Yes	Yes^d^
*NetLogo* (Tisue and Wilensky 2004)	Model‐centric	Repository/Analysis	NetLogo Web^a^	No
*Mathematica* (Wolfram 1991)	Model‐centric	Analysis	No	No
*MATLAB* (MATLAB 2021)	Model‐centric	Analysis	No	No
*Numerus* (Getz et al. 2018)	Model‐centric	Analysis	Export to Web^b^	No
*EBI BioModels* (Le Novere et al. 2006)	Model‐centric	Repository	Not applicable^c^	Yes
*shiny* (R) (Chang et al. 2024)	Model‐centric	Analysis	No	No
*Jupyter* (Kluyver et al. 2016)	Code‐centric	Analysis	No	Yes
*CodeOcean* (Staubitz et al. 2016)	Code‐centric	Code validation	No	Yes

*Note:* We define three broad categories to differentiate between platforms: (i) the ‘approach’ of the platform, whether it is primarily designed for models or for code; (ii) the modelling functionality offered, whether the platform allows model analysis or is a repository and (iii) where computation occurs, which determines what costs and technical hurdles are involved in analysing models.

^a^ *NetLogo* also has a browser‐based version that includes a library of models suitable for running in a browser.

^b^ *Numerus* models can be exported to a format that can be run in a browser.

^c^ *BioModels* contains previously generated plots and is not designed for model analysis.

^d^ By providing an AI‐assisted pipeline for adding models, modelRxiv can accept as input models written in many different frameworks.

To make a repository of models accessible to a broad audience, it is crucial that models can be interacted with easily, without requiring the user to install additional tools or understand the specific programming language used. Indeed, some modelling platforms offer web‐based implementations as an additional feature, where model analysis and visualisation occur in the browser. In this type of web‐based setup, the user is not required to deploy computational containers, making the service sustainable (Table 1). However, these features are not widely used and are often not feasible because most modelling languages lack browser support. The adoption of a platform to act as a repository depends on the majority of models in a field being compatible with it, but the integration of existing models with platforms that are based on specialised programming languages is a substantial challenge. On the other hand, ‘code‐centric’ platforms that support execution of multiple programming languages, such as *Jupyter* (Kluyver et al. 2016) or Code Ocean (Staubitz et al. 2016), often require deployment to cloud resources, making them less ideal to act as a basis for a repository. The code‐centric design of these platforms can also introduce technical hurdles unrelated to understanding the models themselves. Thus, a platform such as modelRxiv that accepts as input models written in different languages, while providing an interface that is designed specifically for modelling, could bridge the gap between model‐centric and code‐centric approaches, making accessible many different types of models in a single repository.

### 3.1 Facilitating Evaluation of Models During and After the Review Process

One of the most important potential applications of modelRxiv is to facilitate and improve the review processes of modelling studies in ecology and evolution. During the review of modelling studies reviewers should, ideally, evaluate the correctness, robustness, reproducibility and applicability of models. However, in practice, even when the model code is attached to the submission, there are technical difficulties including setup, installation of dependencies, compatibility and acquaintance with the specific coding language chosen by the author that makes this time‐consuming and unfeasible in almost all cases. Consequently, models are rarely reproduced by reviewers during the review process and are even more rarely subjected to manipulation and thorough investigation beyond the specific parameter values chosen by the author.

Improving model evaluation during the review process requires development of user‐friendly and accessible tools for reviewers. The understanding that such tools are vital to ensure code validation has led to the adoption of services for deploying data processing code to computational containers by reviewers, such as *CodeOcean* (Staubitz et al. 2016; Cheifet 2021). *CodeOcean* provides an interface through which reviewers can manipulate and deploy code associated with a manuscript during the review process. By removing the technical hurdles of code deployment, code validation can become an integral part of the review process without resulting in a significant additional burden on reviewers.

modelRxiv can facilitate testing model robustness beyond simply validating model code for technical errors. With models uploaded to modelRxiv, reviewers can extensively explore the full parameter space of the model, and can even alter the model itself. Using the presets feature, reviewers could easily generate figures from the manuscript, and assess the robustness of the model in terms of the parameters used to generate these figures. By using presets, the model owner can guide users through stages of exploring the parameter space of the model. This can lead to a more intuitive understanding of the model than a static figure, as the user is free to manipulate the model parameters and observe the effect on the model result, or to visualise model dynamics for different regions within a certain figure.

Making model code validation an integral part of the review process would improve not only confidence in the model results but also encourage more thorough exploration of the model parameter space and the underlying assumptions by the authors. It could also shorten the review process by allowing reviewers to answer queries regarding model parameters without having to rely on the authors to produce additional figures.

As modelRxiv is a public repository, using it in the review process has the important benefit of ensuring that the model would be accessible to readers of the manuscript after it has been published. This may encourage readers of the paper to explore the parameter space of the model beyond what was presented in the manuscript, potentially leading to new insights and directions of research.

### 3.2 Educational Uses

modelRxiv also serves as an educational tool. With models that demonstrate basic principles in ecology and evolution, students can visualise preprepared dynamics and manipulate model parameters to gain intuition on the phenomenon in question, and they can test hypotheses regarding the relationship between model parameters. At a more advanced level, students can explore the code implementation of the model, to generate similar alternative models and to gain experience in model coding and design. The fact that the user is not exposed to the full complexity of the model from the very beginning is an important aspect when encouraging those not familiar with model design or programming to engage in exploration of the model. Therefore, the clear separation of model visualisation and manipulation from the underlying code would be helpful in teaching environments, where it is necessary to account for variable technical abilities of students to provide individualised and more gradual learning curves.

### 3.3 Public Accessibility of Model Results

Beyond facilitating the review process, we believe that modelRxiv could promote and improve the exploration of published models in the ecological and evolutionary disciplines. First, the ease of accessibility to published models would incentivise researchers to expand existing models and utilise existing modelling frameworks, thereby making model development faster. This would also improve the comparability between published models, ensuring that conclusions pertaining to differences between model results can be coherently attributed to changes in key assumptions, rather than to model design or coding. Second, encouraging the scientific community to participate in manipulation of model parameters, in a deeper examination of model parameter spaces, and in adjustment of model assumptions through simple alteration of underlying code, could generate new insights for existing models. Such inquiries and modifications could lead to the identification of novel model behaviours with biological significance, perhaps undetected due to a different focus of the original study. These investigations could also lead to a deeper understanding of the model behaviours, particularly in terms of the boundaries in which the described behaviours of the models no longer hold, and a discussion the biological significance of these boundaries. Therefore, the adoption of modelRxiv by the eco‐evolutionary modelling community could encourage collaborations between researchers to extend and elaborate on modelling studies, for example, between the publishers of the original modelling study and the researchers identifying interesting behaviours in their models.

### 3.4 Limitations

While the modelRxiv platform has many potential applications, some examples of which are listed above, there are also a number of limitations in the current, initial version of the platform. In meeting some of its goals in accessibility, certain limitations in the design of the platform have been introduced that cannot be resolved through code improvements. For instance, enforcing browser computation, so that users do not have to download or install software on their computer, means that models must be implemented in Python or JavaScript. In addition, while modelRxiv can run analyses and utilise multithreading to reduce the time required to generate results, it is currently aimed at models that can produce dynamics on‐the‐fly rather than visualising the results of long‐running analyses. There are additional limitations of the current version that can be improved gradually as more models are added to the platform. For instance, the AI‐assisted model conversion pipeline can be fine‐tuned using previously uploaded models. We can also expand the currently available visualisations and allow more manipulation of these visualisations by users. User feedback on the types of models they would like to upload can also direct the development of future releases, adding features that are necessary to support these models.

## 4 Methods

### 4.1 Implementation of Platform

modelRxiv is browser‐based, and was designed as a serverless application. This greatly reduces operational costs as there is no need to operate a back‐end server, and costs scale with use. These considerations are important to make the project sustainable in the long term as a free, open‐source repository. Further technical details of the implementation are available on the GitHub repository of the project (https://github.com/carrowkeel/modelrxiv).

### 4.2 Model Protocol

To allow modelRxiv to act as a wrapper for models, we developed a protocol with minimal requirements for model integration. This protocol is specifically designed for step‐wise models but will be suitable for any model that generates results through iteration. Model code must implement a ‘step’ function that contains the main model logic, in addition to any other functions that were part of the model code.

In addition to the model code, we designed a model scheme that balances readability by users editing the scheme manually, generation and modification by LLMs, and processing by modelRxiv. The scheme is separated into sections with each section describing a different aspect of the model, including metadata, parameters, plots and presets. The AI‐assisted model uploader can generate model schemes based on code, and attempt to complete or correct partially written schemes.

At present, modelRxiv directly supports code written in Python or JavaScript, as these can be easily executed in a browser environment. However, models written in other languages can be integrated with modelRxiv by using the AI‐assisted conversion pipeline; the simplicity of the model protocol makes this pipeline feasible.

### 4.3 AI‐Assisted Conversion Pipeline

To facilitate the conversion of models from multiple languages to languages compatible with modelRxiv (Python or JavaScript) and to ensure that the code conforms to the modelRxiv protocol, we used general‐purpose commercial LLMs that are not specifically trained on model code. To identify which LLM was most suitable for the specific task of converting models to be compatible with modelRxiv, we tested different instruction sets and OpenAI LLMs (o1‐mini, gpt‐4o and gpt‐4o‐mini). We found that these LLMs performed best when given comprehensive examples of previous models in their instructions. Hence, the instruction consists of the modelRxiv ‘Help’ page (Data S1) and a technical explanation of the task (receiving model code and a scheme and making them compatible with modelRxiv). We then tested the success rates of these instructions on the LLMs mentioned above, for four different programming language inputs: Python, *MATLAB*, *Mathematica* and *R*. For each language, we selected a typical model, in terms of complexity, from those that have been uploaded to modelRxiv. We defined a successful conversion attempt as the LLM providing model code and a scheme that is compatible with modelRxiv and can generate dynamics. We queried each LLM 100 times with the same model to measure the success rate. The results provide a rough estimate of the ability of each LLM to convert different types of models (Table 2). The same evaluation can be conducted with other LLMs; in the current analysis, we used OpenAI LLMs because using the same API simplified the process.

**TABLE 2:** Success rates of model conversion using different OpenAI LLMs.

LLM	Python	*MATLAB*	*Mathematica*	*R*
gpt‐4o‐mini*	0.17	0.32	0.44	0.81
gpt‐4o	0.51	0.50	0.74	0.96
o1‐mini	0.13	0.72	0.95	0.94

*Note:* LLMs were tested by asking them to convert a model to be compatible with modelRxiv in a single attempt (100 repeats). We tested four programming languages: Python, *MATLAB*, *Mathematica* and *R*. For each language, we selected a typical model, in terms of complexity, from those that have been uploaded to modelRxiv. A successful conversion attempt was defined as the output model code and scheme being compatible with modelRxiv and able to generate dynamics. The LLMs are in ascending order of complexity. *For gpt‐4o‐mini, unlike for the other two LLMs, we generated the scheme and code separately because it showed inferior results when attempting to convert both in a single interaction.

Of the tested LLMs, o1‐mini had consistently high scores for all programming language inputs expect for Python (Table 2). o1‐mini is expected to have higher scores for advanced reasoning based on statistics provided by OpenAI (2024). Its inferior performance with Python code appeared to be owing to deviation from the instructions provided to the LLM. This result emphasises the fact that more advanced LLMs are not necessarily better at all tasks compared to simpler ones, a fact that should be taken into consideration in future developments of such platforms.

This analysis is presented only as an indication of relative LLM suitability for this specific task. Specifically, we did not consider the accuracy of the visualisation produced by the converted model, and a successful conversion attempt was defined only as the model code and scheme being compatible with modelRxiv. On the other hand, the modelRxiv upload interface allows users to query the conversion pipeline multiple times if the returned code has errors, or if the visualisation of dynamics is inaccurate. In subsequent conversion attempts, the LLM will have access to any errors that occurred when the model was run, in addition to feedback from the user regarding visualisation inaccuracies.

## 5 Accessibility of Data

The modelRxiv platform and all models presented here are freely accessible at https://modelrxiv.org. The platform code is available at github.com/carrowkeel/modelrxiv and github.com/carrowkeel/apc. A static version of the platform code is available at https://doi.org/10.5281/zenodo.14229154. All platform code is licensed under AGPLv3. Models are licensed under CC‐BY 4.0 unless otherwise stated. We presented results for the OpenAI LLMs o1‐mini, gpt‐4o and gpt‐4o‐mini (Table 2). The OpenAI LLM used for model conversion in the current version of modelRxiv is o1‐mini. To allow users to provide feedback on bugs and issues using modelRxiv, we opened a *Slack* workspace that can be joined using the invitation link on the ‘contribute’ page (https://modelrxiv.org/contribute.html).

## Author Contributions

Keith D. Harris designed and developed the platform. Gili Greenbaum supervised the development of modelling features, and Guy Hadari consulted on cloud integration. Keith D. Harris and Gili Greenbaum wrote the manuscript. All authors read and approved the final version of the manuscript.

## Supporting information


Data S1.

## Data Availability

The modelRxiv platform and all models presented here are freely accessible at https://modelrxiv.org. The latest platform code is available at github.com/carrowkeel/modelrxiv and github.com/carrowkeel/apc. A static version of the platform code is available at https://doi.org/10.5281/zenodo.14229154. All platform code is licensed under AGPLv3. Models are licensed under CC‐BY 4.0 unless otherwise stated. We presented results for the OpenAI LLMs o1‐mini, gpt‐4o and gpt‐4o‐mini (Table 2). The OpenAI LLM used for model conversion in this version of modelRxiv is o1‐mini.
