# Supplementary material for: modelRxiv: A Platform for the Dissemination and Interactive Display of Models
Source: Ecol Lett. 2024 Dec 31;28(1):e70042. doi: 10.1111/ele.70042 (PMC11687343; doi:10.1111/ele.70042)
Supplement: Supplementary file 1 — Data S1. [file ELE-28-0-s001.pdf]

Uploading models to modelRxiv

This page provides an overview of the process of uploading models, including details about implementing the modelRxiv protocol. Researchers are encouraged to upload their published or unpublished models to modelRxiv to allow a wider audience to access the model, manipulate parameters, and regenerate results. modelRxiv does not require that uploaded models be accepted at a peer-reviewed journal before uploading, but you must be allowed to make the model code publicly available (see our terms of use for more information. To read more about the goals of the platform, see the preprint.

Registration

Before uploading a model, you will need to create an account and log in. This can be done by clicking on the "Login" button on the top right corner of the page, and opening the "Register" tab. Once you have created an account, you can login using your credentials on the Login dialog box.

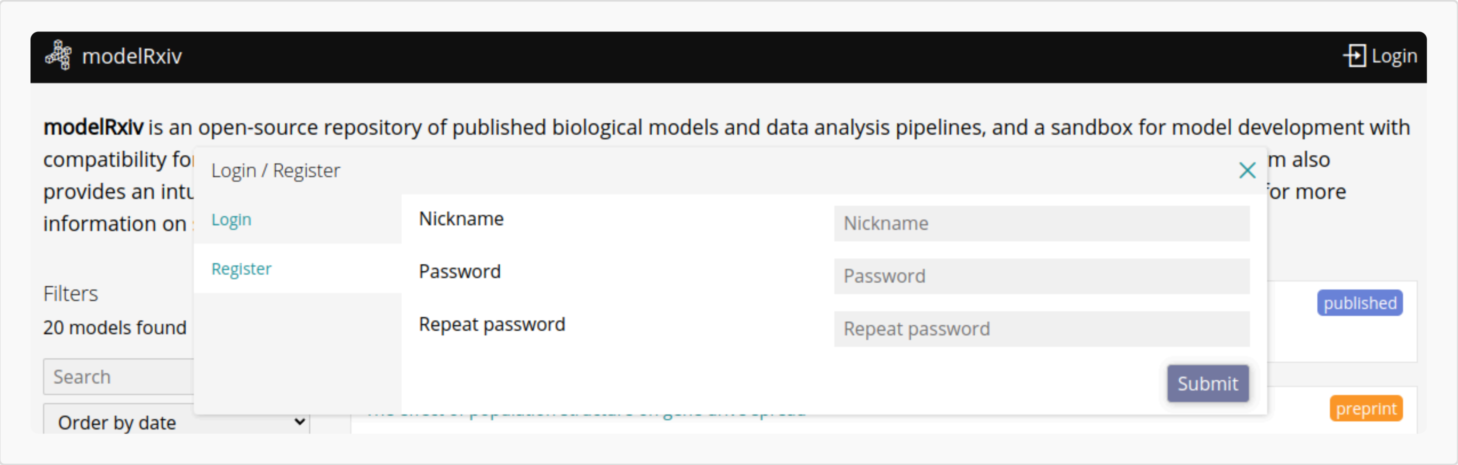

Once logged in, you will see an "Upload" button at the top right corner of the page.

Upload page

The upload page is designed to allow uploaders to paste their model code and gradually adapt it to modelRxiv, either by manually editing the code and testing it, or by using the AI-assisted features for code conversion. The upload process has four main steps:

- Adding your model code. The model code box must contain the entire code necessary to generate the model results and be compatible with modelRxiv (see below).
- Describing your model using the modelRxiv scheme format. The scheme includes publication information, the list of parameters that users can manipulate, visualizations of the dynamics, and presets of parameters that allow users to regenerate certain scenarios or figures.
- An optional step of using the AI-assisted conversion pipeline to make your model compatible with modelRxiv.
- Testing your model to assure that it works and is compatible with modelRxiv. Once you have successfully tested the model, it can be uploaded.

Model code

When you first open the upload page, you will see a blank text box for your code. You can either provide code that conforms to the modelRxiv protocol, or use the AI-assisted pipeline to convert it (recommended). To see an example of a simple model that is compatible with modelRxiv to use as a template, click the "Load example" button at the bottom right of the text box.

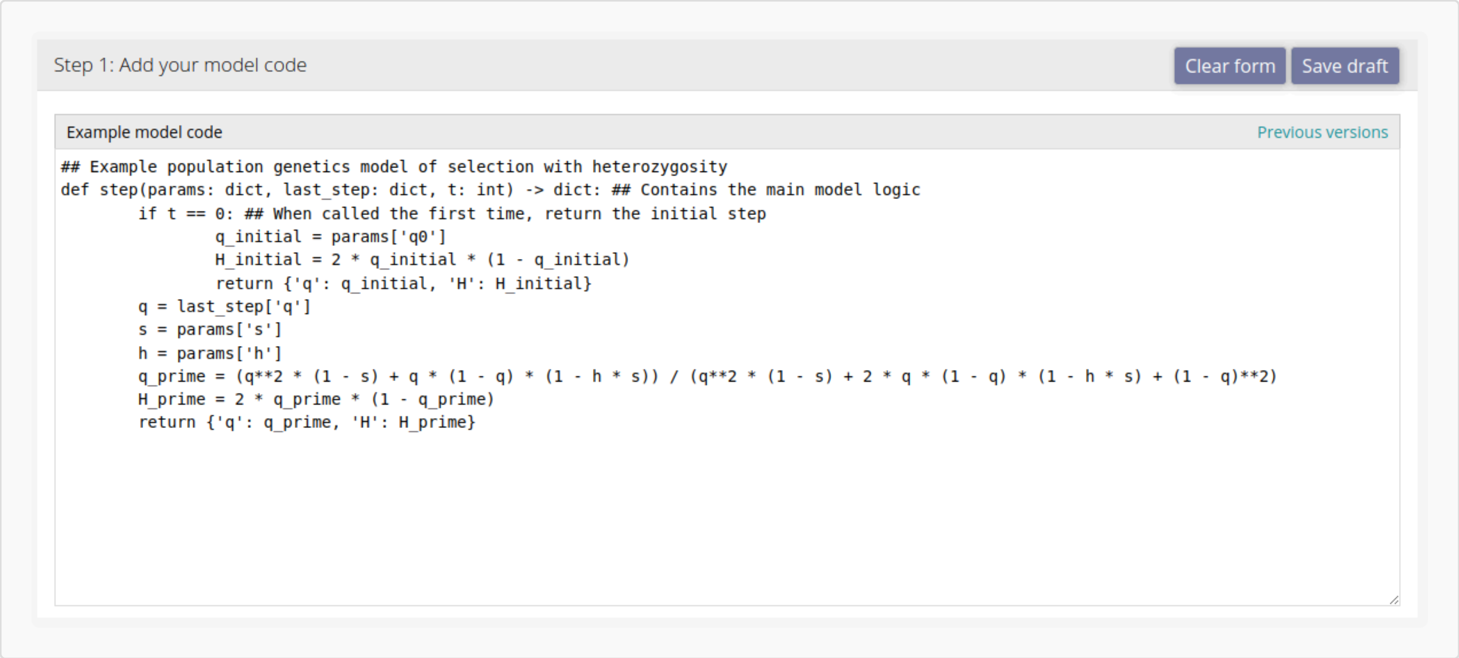

Model scheme

The model scheme describes your model to modelRxiv, including its parameters and visual outputs. This allows modelRxiv to provide the appropriate adjustable parameters to users, and to create dynamic plots based on the output of running the model code. You can also add descriptions to the scheme to help users understand your model.

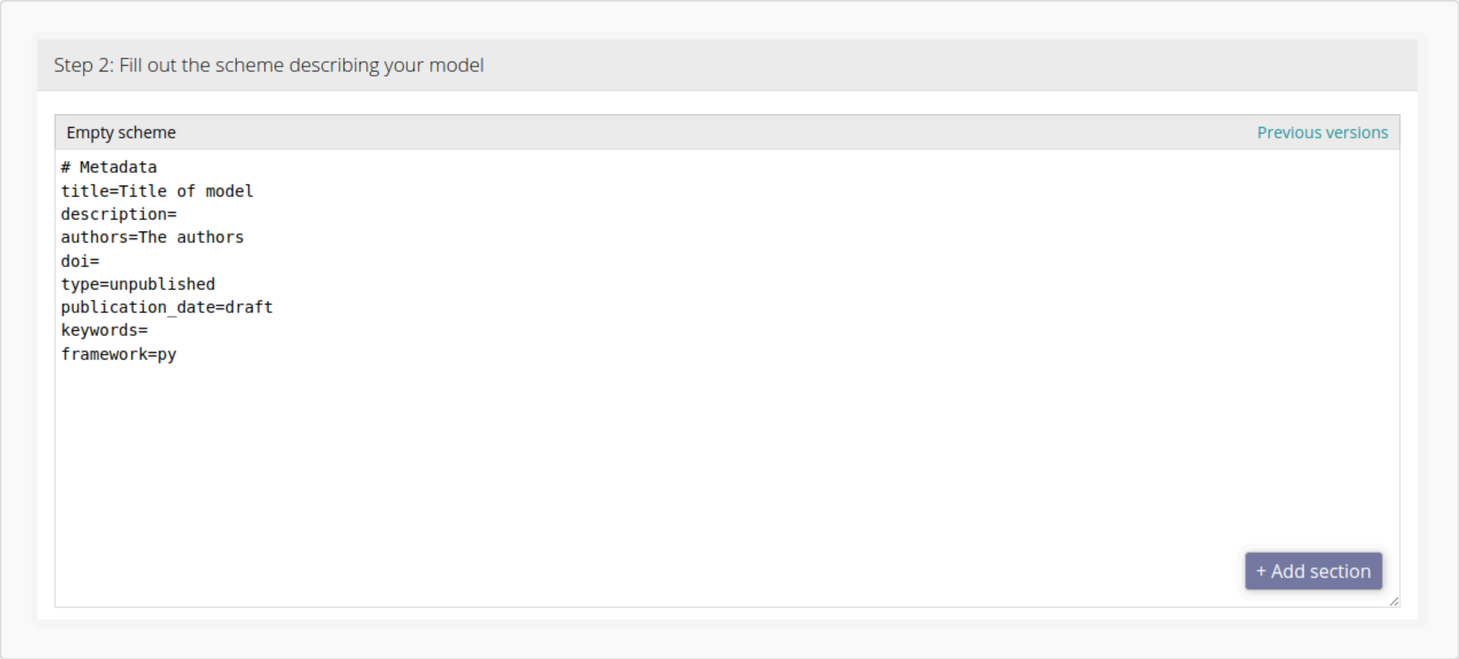

Example model

To demonstrate the scheme, we consider a simple model of selection:

```python
def step(params: dict, last_step: dict, t: int) -> dict:
    # Compute the next step given the last step and the model parameters
    if t == 0: # When called the first time, return the initial frequency
        return {'q': params['q0']}
    q = last_step['q']
    s = params['s']
    h = params['h']
    q_prime = (q**2 * (1 - s) + q * (1 - q) * (1 - h * s)) / (q**2 * (1 - s) + 2 *
q * (1 - q) * (1 - h * s) + (1 - q)**2)
    return {'q': q_prime}
```

The scheme is divided into sections: each section begins with a line starting with a hash character `#` followed by the type of section. The following lines are parameter definitions for the specific section.

The basic section of the scheme describes the study and possibly the publication to which the model relates. For instance, in the example below, the scheme metadata is filled with information relating to a specific publication:

```model scheme
# Metadata
title=The Genetical Theory of Natural Selection
description=Natural Selection is not Evolution. Yet, ever since the two words have
been in common use, the theory of Natural Selection has been employed as a
convenient abbreviation for the theory of Evolution by means of Natural Selection,
put forward by Darwin and Wallace. This has had the unfortunate consequence that
the theory of Natural Selection itself has scarcely ever, if ever, received
separate consideration.
authors=R. A. Fisher
doi=
type=published
publication_date=1930
keywords=natural-selection,evolution
framework=py
```

Note that the metadata also includes the field "framework", which indicates which coding framework the model code uses. This can either be "py" for Python or "js" for JavaScript.

Now we want to allow a parameter in the model to be "exposed" to the user, so that they can manipulate the parameter using the interface. We can do this by adding a "Parameter" section:

```model scheme
# Parameter: s
name=s
```

```
description=The relative fitness cost of the allele
value=0.1

# Parameter: Steps
name=step_num
description=Number of steps to run the model for
value=100
```

This adds a parameter `s` with a default value `0.1` to the model, with the label "s". The label can also contain an underscore character `_` to use subscript formatting. We also define a parameter `step_num`, which is a special parameter as it tells modelRxiv for how many steps to run the model. By default (without defining the parameter in the scheme), its value is 100.

Now that we have some model parameters, we can add plots. Plots take the output of the model code and connect them to a specific plot type. In the example below, we take the frequency of the allele from the model, the dynamic parameter `q`, and create a line plot for `q` over the time steps of the model:

```
model scheme

# Plot: Allele frequency over time
type=line
x=t
y=q
xlabel=Generations
ylabel=Frequency
```

In this example, we set the x-axis as `t`, which is the time step of the model, and the y-axis as `q`. We also added x- and y-axis labels. We could additionally set the x- and y-axis limits, for instance, using `xlim=0,0.5`. For available plots and parameters, see the list of visualizations.

Optional scheme features

In many cases it is helpful to guide users through specific scenarios of interest by providing parameter presets. If you provide presets with your model, they will be displayed to the user by default, instead of the list of parameters. They can then choose specific presets (which will run the model with the specific parameter set), and begin exploring the model by modifying the parameters around these scenarios of interest. Here are two example presets in our simple evolutionary model:

```
model scheme

# Preset: Positive selection, recessive
q0=0.1
s=-0.1
h=0

# Preset: Negative selection, dominant
q=0.5
```

```
s=0.1
h=1
```

You can add as many parameter definitions as you like to each preset. These will modify the existing user parameters and run the model, when clicked by a user.

AI-assisted conversion pipeline

The conversion pipeline was designed to reduce the effort required for integrating existing models with modelRxiv. It takes the model code and the current scheme text and attempts to convert the model code so that it is compatible, and create a scheme that matches parameters and plots described in the model code. By using this pipeline, you consent to the original model code being sent to servers of the LLM provider, and being retained on our servers for the purpose of improving our pipeline (see our privacy policy for more details).

Below is an example of the text response of the AI-assisted conversion pipeline. Under the response is a comments box where you can add additional comments for the conversion pipeline. By default, any errors that appear while testing the model (see below) will be passed to the conversion pipeline. The comments box allows you to add additional comments, such as adjustments of the visualization of the dynamics, that will be sent to the conversion pipeline along with the code, scheme and any errors.

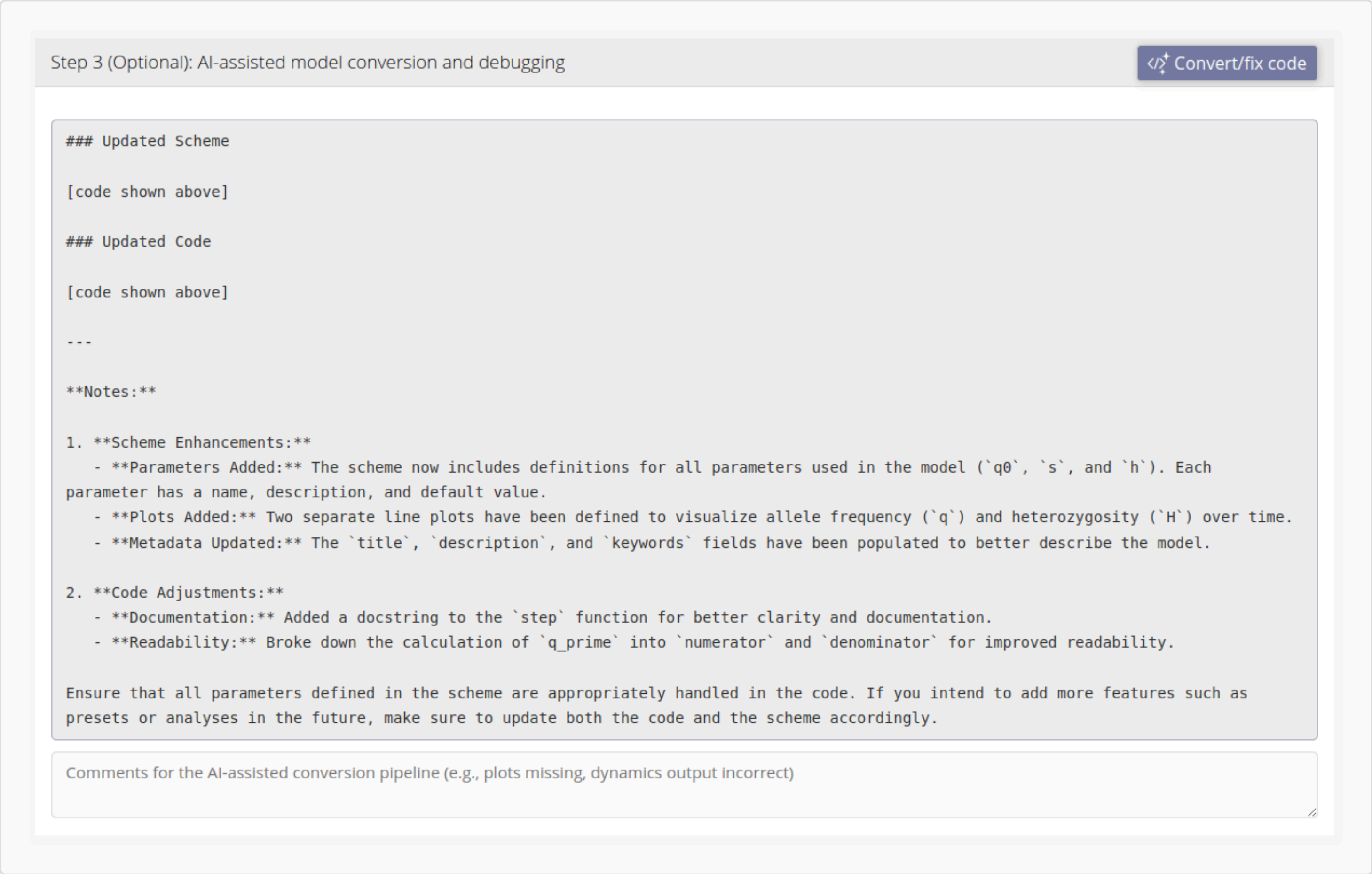

Testing your model

At any stage of uploading your model you can run a test to see that modelRxiv is correctly interpreting the scheme and producing the correct dynamics output. Errors that occur while attempting to run the model will be displayed in an error box in this section of the form. Here we test the example model we discussed above and generate model dynamics:

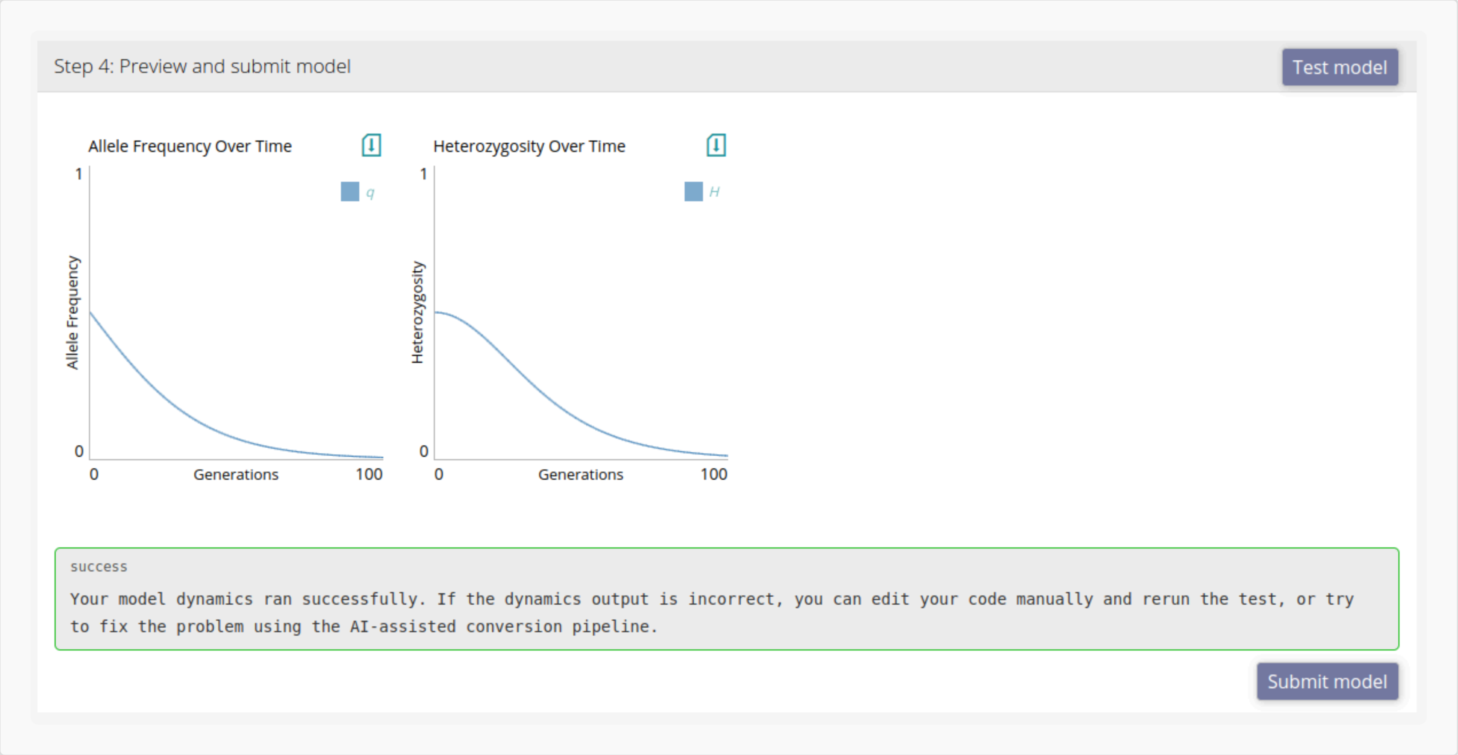

The model passed the test, and we can now upload the model by clicking "Submit". If there is a problem with the dynamics, we can either fix the code and scheme manually, or add a comment in the comments box within the AI-assisted conversion pipeline section of the upload form (as explained in the section above). If you receive an error when testing the model, clicking "Convert/fix" will send the error together with any comments you added to the conversion pipeline.

Model analyses

modelRxiv is aimed at models that can generate dynamics visualizations on-the-fly rather than running computationally intensive analyses that might take hours or days. However, seeing as models may include results that do not fit the step-wise dynamics display, modelRxiv also allows uploaders to specify a function in their code that can generate a figure. Analyses run some code and return an image, or data that matches one of the visualizations on modelRxiv. For instance, we can use the `matplotlib` library to generate an image that will be displayed in the browser:

```
python
import matplotlib.pyplot as plt

async def my_analysis(params):
    fig = plt.figure(figsize=(6,6)) # Create a square image
    plt.imshow(np.random.normal(size=(100,100), scale=params['s'], loc=0.5))
    plt.axis('off') # Remove axes
```

```
    plt.tight_layout(pad=0) # Remove padding around image
    plt.show()
```

In this example, we intentionally hide the axes and remove padding so that the image fits the square grid on modelRxiv. We can then specify the axes in the analysis definition in the model scheme:

```
model scheme

# Analysis: generate_grid
function=my_analysis
type=image
xlabel=x
ylabel=y
xlim=0,100
ylim=0,100
```

In the example below, we define an analysis that generates a 2-dimensional grid figure.

```
model scheme

# Analysis: fixation_analysis_grid
function=run_fixation_grid
type=mat_grayscale
xlabel=s
ylabel=h
xlim=0,1
ylim=0,1
```

Note that the function `run_fixation_grid` must be defined in your code and return data that fits the `mat_grayscale` (grayscale matrix) plot. Below is an example of such a function that runs the model dynamics and returns a result of 0 or 1 for a grayscale matrix.

```
python

# This function runs the model and returns 1 if the allele is close to fixation
async def single_run(params):
    last_step = None
    for t in range(0, int(params['step_num']) + 1):
        next_step = step(params, last_step, t)
        if not next_step:
            break
        last_step = next_step
    return int(last_step['q'] > 0.99)

async def run_fixation_grid(params):
    # Create a grid of parameters, this could also be done with loops
    s = np.linspace(0, 1, 51)
    h = np.linspace(0, 1, 51)
    grid_x, grid_y = np.meshgrid(s, h)
    grid = np.array([grid_x.reshape(len(s) * len(h)), grid_y.reshape(len(s) *
```

```
len(h))]).T
    # Combine with input parameters
    param_sets = [{**params, 's': x_y[0], 'h': x_y[1]} for x_y in grid]
    # Distribute grid computation
    results = await distribute('single_run', param_sets)
    normalized_matrix = np.array(results).reshape(len(s), len(h)).tolist()
    return normalized_matrix
```

Note that we used the `distribute` function in order to compute the model results in parallel. This is handled by modelRxiv and ensures that computational resources are not overused by multiple analyses.

Now that we defined this analysis, we can also define presets that run it with different fixed parameters:

```
model scheme

# Preset: Fig. 2: Fixation grid (q0=0.5)
analysis=fixation_analysis_grid
q0=0.5

# Preset: Fig. 2: Fixation grid (q0=0.1)
analysis=fixation_analysis_grid
q0=0.1
```

When users click these presets, the grid will be computed by running the `run_fixation_grid` in your code. Users will see a progress bar on the preset while the result is being generated. We suggest avoiding analyses that take more than a few minutes to compute results (even with multi-threading), as users will likely navigate away from the page if the waiting time is too long.

Visualizations

modelRxiv currently supports a number of plots for dynamics data. These plots are listed below. For analyses, it is possible to use the Python library matplotlib; figures generated in analysis functions using matplotlib (see the example above) will appear in the browser as images.

Line plot (`line`)

This is the most common plot for time-series data. If the x-axis is set as `t`, which each step it updates one or more lines in the plot using the value of `y`.

```
model scheme                                                    show screenshot

# Plot: Population size over time
type=line
x=t
y=N
xlabel=Generations
ylabel=Population size
```

```
xlim=0,step_num
ylim=0,K
```

In this example, we also set limits for the axes using `xlim` and `ylim`. These fields take two numbers or parameter names separated by a comma. For `xlim`, we used `0,step_num`, meaning that the x-axis goes from 0 to the value of the `step_num` parameter, which is the parameter that tells modelRxiv how many iterations to run the dynamics for. For `ylim`, we used `0,K`, meaning that the y-axis goes from 0 to the value of a carrying capacity parameter `K`. This means that if the user updates these parameters, the plot limits will be updated. A step function that produces data for this plot might look like:

```python
def step(params, last_step, t):
    K = params['K']
    r = params['r']
    if not last_step:
        return {'N': params['K'] / 2} # Set initial population size
    N = last_step['N']
    N_next = r * N * (1 - (N / K))
    return {'N': N_next}
```

To be compatible with the line plot, `N` should be a number (it can be an int or a float). Note that while we need to return `N` for the dynamics plot, `t` does not need to be returned as it is provided to the dynamics plot by modelRxiv.

To plot multiple lines, for instance if we had multiple populations, you can use a comma to separate the names of the output parameters. In the example below, we also use the `legend` parameter to specify how the lines will be annotated (with indices as subscript).

model scheme — show screenshot

```
# Plot: Population size over time
type=line
x=t
y=N1,N2
xlabel=Generations
ylabel=Population size
xlim=0,step_num
ylim=0,K
legend=N_1,N_2
```

Multiple line plot (`lines`)

This plot is designed for plotting lines that represent replicates in a stochastic simulation. For instance, it could be used to track allele frequency of separate simulations of a drift model:

```
model scheme                                        show screenshot

# Plot: Allele frequency in repeats
type=lines
xlabel=Generations
ylabel=Frequency
xlim=0,step_num
ylim=0,1
x=t
y=q
```

The y-axis data includes the `q` parameter. To be compatible, `q` should be an array of allele frequencies. Lines will be shown with low opacity, with one line representing the mean of all repeats with high opacity. Here is an example `step` function that returns values compatible with this plot:

```python
def step(params, last_step, t):
    repeats = params['repeats']
    N = params['N']
    if not last_step:
        return {'q': np.full(repeats, 0.5)} # Start all simulations at q=0.5
    q = np.array(last_step['q'])
    q_next = np.random.binomial(N, q, size=len(q)) / N # Drift
    return {'q': q_next}
```

Scatter plot (`scatter`)

This plot is useful for dynamically displaying data with two dimensions, such as the position of objects moving in space, or the correlation between two attributes of an agent. In the example below, we track the movement of two overlapping populations in 2-dimensional space.

```
model scheme                                        show screenshot

# Plot: Population movement
type=scatter
xlim=0,100
ylim=0,100
data=pop1,pop2
```

The inputs `pop1` and `pop1` are arrays containing the 2-dimensional coordinates of the agents in each population. Note that we use `data` instead of `x` and `y` to provide data to the plot: output provided using `data` will replace the contents of the plot with each time-step. Below is example code that works with the above scheme:

```python
def step(params: dict, last_step: dict, t: int) -> dict:
    N = params['N']
```

```
    if t == 0:
        # Random initial coordinates
        pop1 = np.random.choice(100, (N, 2))
        pop2 = np.random.choice(100, (N, 2))
        return {'pop1': pop1, 'pop2': pop2}
    # Update agent coordinates using random steps multipled by step size
    pop1_next = np.array(last_step['pop1']) + np.random.choice([-1, 1], size=(N, 
2)) * params['move_size_1']
    pop2_next = np.array(last_step['pop2']) + np.random.choice([-1, 1], size=(N, 
2)) * params['move_size_2']
    return {'pop1': pop1_next, 'pop2': pop2_next}
```

2-dimensional matrix (`mat`)

This plot is useful for visualizing patches. For instance, it could visualize changes across a landscape or population structure. In this example, we provide a matrix of alleles frequencies `q` directly to the plot using `data`. The values of the cells in the matrix (normalized to between 0 and 1) will determine the color of the patch. Not that the x- and y-axis limits here are for display only.

```
model scheme                                                              show screenshot

# Plot: Patches
type=mat
xlim=0,1
ylim=0,1
data=q
legend=Low frequency,High frequency
```

This plot is the equivalent of matplotlib's `imshow`. The x- and y-axis limits are provided here for display only since the input data does not contain coordinates. A grayscale equivalent of this plot is `mat_grayscale`. Below is an example model of gene flow and drift in a stepping-stone population structure, which uses the `mat` plot above to visualize the distribution of allele frequencies across patches:

```
python

def migration_matrix_from_edge_matrix(params, edge_matrix):
    n = edge_matrix.shape[0]
    M = edge_matrix * params['m']
    M[range(n), range(n)] = 0
    M[range(n), range(n)] = 1 - np.sum(M, axis=1)
    return M

def generate_graph_from_grid(grid_size):
    mat_size = grid_size**2
    M = np.zeros([mat_size, mat_size])
    main_diag = np.diag_indices(mat_size, 2)
    d1 = np.array([main_diag[0][:-1], main_diag[1][1:]]).T
    d2 = np.array([main_diag[0][:-grid_size], main_diag[1][grid_size:]]).T
```

```
    M[d1[:,0], d1[:,1]] = 1
    M[d2[:,0], d2[:,1]] = 1
    M[d1[:,0][grid_size-1::grid_size], d1[:,1][grid_size-1::grid_size]] = 0
    return M + M.T

def step(params, last_step, t):
    grid_size = params['grid_size']
    if not last_step:
        q = np.full((grid_size, grid_size), 0.5)
        return {'q': q, 'patches': np.zeros((0, 3))}
    edge_matrix = generate_graph_from_grid(grid_size)
    M = migration_matrix_from_edge_matrix(params, edge_matrix)
    q_migration = np.dot(np.array(last_step['q']).reshape(grid_size**2), M)
    q_drift = np.random.binomial(params['N'], q_migration, size=len(q_migration)) / 
params['N']
    q_matrix = q_drift.reshape(grid_size, grid_size)
    not_fixed_indices = np.where((q_matrix > 0.01) & (q_matrix < 0.99))
    patches = np.array([*not_fixed_indices, q_matrix[not_fixed_indices]]).T
    return {'q': q_matrix, 'patches': patches}
```

2-dimensional grid (`grid`)

If you want to specify the coordinates of the patches (potentially not filling the entire grid) you can use `grid` instead. The input must now specify the coordinates and the value of patches, and the x- and y-axis limits in the scheme will determine the position of the data points. For the scheme we provide `patches` instead of `q`. We provided the `patches` output that fits this plot in the code above. To demonstrate, we display only patches that are not fixed for either allele.

```
model scheme                                                          show screenshot

# Plot: Patches without fixed
type=grid
xlabel=x
ylabel=y
xlim=0,grid_size
ylim=0,grid_size
data=patches
legend=Low frequency,High frequency
```

Here `patches` is an array of 3-dimensional vectors, the same as the input of `nodes` for the `network_plot` plot (i.e., each element of the array contains the coordinates of the patch and its value). As with `network_plot`, the x- and y-axis here scale the data, so it is important that they match the actual limits of the coordinates provided.

2-dimensional network (`network_plot`)

This plot is useful for representing a network with nodes and edges that updates at every time step, to visualize the distribution of change in nodes across the network. Nodes have coordinates and a value

between 0 and 1 that defines their color. This can be used to visualize various network structures; here we repeat the example from above (in the `mat` plot) to demonstrate its use.

```
model scheme                                                    show screenshot

# Plot: Population structure
type=network_plot
xlabel=x
ylabel=y
xlim=0,1
ylim=0,1
data=nodes_edges
legend=Low frequency,High frequency
```

We use the `nodes_edges` output of the model for the plot, which contains the position of the nodes in the grid and their value (in this case, their allele frequency), and the edges between nodes. We can also indicate what the extremes of the color scale indicate using the `legend`. Importantly, the x- and y-axis limits should be set according to the maximal coordinate values of the nodes. The example code below is the same as for the `grid` plot, only that now we also specify edges to be draw on the plot.

```python
def migration_matrix_from_edge_matrix(params, edge_matrix):
    n = edge_matrix.shape[0]
    M = edge_matrix * params['m']
    M[range(n), range(n)] = 0
    M[range(n), range(n)] = 1 - np.sum(M, axis=1)
    return M

def lines_from_edge_matrix(edge_matrix, nodes):
    if len(nodes) == 0:
        return []
    indices = np.triu_indices(edge_matrix.shape[0], k=1)
    edges = np.array(indices).T[edge_matrix[indices] > 0.0001]
    lines = nodes[edges]
    return lines

def generate_graph_from_grid(grid_size):
    mat_size = grid_size**2
    M = np.zeros([mat_size, mat_size])
    main_diag = np.diag_indices(mat_size, 2)
    d1 = np.array([main_diag[0][:-1], main_diag[1][1:]]).T
    d2 = np.array([main_diag[0][:-grid_size], main_diag[1][grid_size:]]).T
    M[d1[:,0], d1[:,1]] = 1
    M[d2[:,0], d2[:,1]] = 1
    M[d1[:,0][grid_size-1::grid_size], d1[:,1][grid_size-1::grid_size]] = 0
    nodes = 0.5 / grid_size + np.indices((grid_size,
grid_size)).T.reshape(grid_size**2, 2) / grid_size
    return M + M.T, nodes
```

```
def step(params, last_step, t):
    edge_matrix, nodes = generate_graph_from_grid(params['grid_size'])
    edges = lines_from_edge_matrix(edge_matrix, nodes)
    M = migration_matrix_from_edge_matrix(params, edge_matrix)
    if not last_step:
        q = np.full(M.shape[0], 0.5)
        nodes_q = np.insert(nodes, 2, q, axis=1)
        return {'q': q, 'nodes_edges': [nodes_q, edges]}
    q_migration = np.dot(last_step['q'], M)
    q_drift = np.random.binomial(params['N'], q_migration, size=len(q_migration)) /
params['N']
    nodes_q = np.insert(nodes, 2, q_drift, axis=1)
    return {'q': q_drift, 'nodes_edges': [nodes_q, edges]}
```

Feedback

This help page will gradually expand as we develop more types of plots, and improve the clarity and flexibility of the model scheme. You can provide feedback about modelRxiv features by sending an email to help@modelrxiv.org.
